# Supplementary material for: Interferon lambda 4 impacts the genetic diversity of hepatitis C virus
Source: eLife. 2019 Sep 3;8:e42463. doi: 10.7554/eLife.42463 (PMC6721795; doi:10.7554/eLife.42463)
Supplement: Supplementary file 1. [file elife-42463-supp1.docx]

**Supplementary File 1:** Demographic, clinical and genetic characteristics of the BOSON and EAP cohorts.

| Cohort | EAP | BOSON |
| --- | --- | --- |
| Number | 74 | 411 |
| Female | 14 (18.9%) | 131 (31.9%) |
| Age (mean, years) | 54.92 | 50.1 |
| Cirrhosis | 74 (100%) | 135 (33%) |
| HCV Viral Load (mean, IU/ml) | 837654 | 4535217 |
| rs12979860 CC | 41 (55.4%) | 147 (35.8%) |
| rs12979860 CT | 26 (35.1%) | 207 (50.3%) |
| rs12979860 TT | 7 (9.5%) | 57 (14.9%) |
| minor allele frequency |  |  |
| rs12979860 (T) | 0.27 | 0.37 |
| rs368234815 (ΔG) | 0.27 | 0.37 |
| rs117648444 (A) | 0.07 | 0.07 |
| Linkage disequilibrium between rs12979860 and rs368234815 (r2) | 1 | 0.98 |
