# Supplementary material for: Interferon lambda 4 impacts the genetic diversity of hepatitis C virus
Source: eLife. 2019 Sep 3;8:e42463. doi: 10.7554/eLife.42463 (PMC6721795; doi:10.7554/eLife.42463)
Supplement: Supplementary file 2. — We used logistic regression to test for association between host IFNL4 SNP (CC vs. non-CC) and presence and absence of amino acids. We included the first two viral and the first three host PCs as covariates. Only amino acids that were present in at least 20 samples were tested (977 amino acids in 471 sites). For each associated site, we have reported all amino acid with a count of >= 20 in reducing frequency order and highlighted the most associated amino acid in bold. The amino acid frequency in CC and nonCC patients, the P-value, log(OR), the standard error and q-value are reported for the most associated amino acid at the site. [file elife-42463-supp2.docx]

**Supplementary File 2:** Host IFNL4 SNP rs12979860 association with HCV amino acids at 10% FDR. We used logistic regression to test for association between host IFNL4 SNP (CC vs. non-CC) and presence and absence of amino acids. We used the first two viral and the first three host PCs as covariates. Only amino acids that were present in at least 20 samples were tested (977 amino acids in 471 sites). For each associated site, we have reported all amino acid with a count of >=20 in reducing frequency order and highlighted the most associated amino acid in bold. The amino acid frequency in CC and nonCC patients, the p-value, log(OR), the standard error and q-value are reported for the most associated amino acid at the site.

| HCV amino acid position | HCV Gene | Amino acids with count >= 20 in reducing frequency order | Frequency of the most associated amino acid in CC genotype | Frequency of the most associated amino acid in non-CC genotype | P-value of the most associated amino acid | Log OR | Standard error | q value |
| --- | --- | --- | --- | --- | --- | --- | --- | --- |
| 2570 | NS5B | **V**AT | 0.239 | 0.51 | 1.30E-08 | 1.19 | 0.21 | 8.20E-06 |
| 2414 | NS5A | **S**NG | 0.62 | 0.832 | 2.10E-07 | 1.15 | 0.22 | 4.80E-05 |
| 2991 | NS5B | H**Y** | 0.503 | 0.261 | 2.50E-07 | -1.04 | 0.2 | 4.80E-05 |
| 2266 | NS5A | A**T**V | 0.303 | 0.115 | 3.20E-07 | -1.26 | 0.25 | 4.80E-05 |
| 576d | E2 | **E**GD | 0.39 | 0.595 | 6.40E-06 | 0.89 | 0.2 | 5.70E-04 |
| 60 | C | G**E** | 0.033 | 0.185 | 1.00E-05 | 2 | 0.45 | 7.80E-04 |
| 372 | E1 | A**T**V | 0.149 | 0.034 | 1.80E-05 | -1.67 | 0.39 | 1.30E-03 |
| 2937 | NS5B | **K**R | 0.79 | 0.919 | 2.10E-05 | 1.22 | 0.29 | 1.30E-03 |
| 940 | NS2 | **S**N | 0.947 | 0.814 | 8.40E-05 | -1.43 | 0.36 | 4.30E-03 |
| 1825 | NS4B | **S**AG | 0.654 | 0.684 | 9.00E-05 | 1.36 | 0.35 | 4.30E-03 |
| 109 | C | **P**Q | 0.849 | 0.959 | 1.40E-04 | 1.38 | 0.36 | 5.50E-03 |
| 521 | E2 | **A**VRIS | 0.471 | 0.65 | 1.50E-04 | 0.73 | 0.19 | 5.50E-03 |
| 501 | E2 | S**N**TDK | 0.42 | 0.261 | 1.90E-04 | -0.76 | 0.2 | 6.50E-03 |
| 1416 | NS3 | A**E** | 0.128 | 0.037 | 2.40E-04 | -1.4 | 0.38 | 7.80E-03 |
| 578 | E2 | **D**H | 0.697 | 0.834 | 4.10E-04 | 0.81 | 0.23 | 1.30E-02 |
| 2088 | NS5A | N**S**D | 0.037 | 0.145 | 4.50E-04 | 1.49 | 0.42 | 1.30E-02 |
| 1740 | NS4B | **T**S | 0.846 | 0.936 | 7.10E-04 | 1.08 | 0.32 | 1.90E-02 |
| 741 | E2 | **I**V | 0.781 | 0.902 | 8.10E-04 | 0.9 | 0.27 | 2.10E-02 |
| 479 | E2 | **G** | 0.84 | 0.935 | 9.00E-04 | 1.04 | 0.31 | 2.10E-02 |
| 72 | C | **E**D | 0.891 | 0.972 | 9.40E-04 | 1.43 | 0.43 | 2.10E-02 |
| 349 | E1 | **A**TV | 0.809 | 0.916 | 9.50E-04 | 0.93 | 0.28 | 2.10E-02 |
| 2268 | NS5A | L**P** | 0.101 | 0.027 | 1.10E-03 | -1.43 | 0.44 | 2.30E-02 |
| 457 | E2 | **S** | 0.828 | 0.929 | 1.10E-03 | 1 | 0.31 | 2.30E-02 |
| 402 | E2 | **L**F | 0.733 | 0.861 | 1.10E-03 | 0.78 | 0.24 | 2.30E-02 |
| 2034 | NS5A | S**T** | 0.138 | 0.276 | 1.20E-03 | 0.82 | 0.25 | 2.40E-02 |
| 2567 | NS5B | **V**M | 0.893 | 0.966 | 1.40E-03 | 1.3 | 0.41 | 2.70E-02 |
| 1158 | NS3 | L**I** | 0.037 | 0.148 | 1.60E-03 | 1.38 | 0.44 | 2.90E-02 |
| 399 | E2 | F**L**IV | 0.317 | 0.454 | 1.90E-03 | 0.62 | 0.2 | 3.10E-02 |
| 929 | NS2 | **M**TV | 0.601 | 0.463 | 1.90E-03 | -0.6 | 0.19 | 3.10E-02 |
| 2263 | NS5A | TP**A**D | 0.048 | 0.131 | 2.40E-03 | 1.18 | 0.39 | 3.50E-02 |
| 2940 | NS5B | **I**T | 0.724 | 0.831 | 2.40E-03 | 0.7 | 0.23 | 3.50E-02 |
| 524 | E2 | A**V**T | 0.481 | 0.348 | 2.50E-03 | -0.6 | 0.2 | 3.50E-02 |
| 1975 | NS5A | **D** | 0.904 | 0.973 | 2.80E-03 | 1.32 | 0.44 | 3.70E-02 |
| 1962 | NS4B | QR**K** | 0.037 | 0.114 | 2.80E-03 | 1.29 | 0.43 | 3.70E-02 |
| 2371 | NS5A | P**S** | 0.075 | 0.02 | 2.90E-03 | -1.51 | 0.51 | 3.70E-02 |
| 1202 | NS3 | S**N** | 0.133 | 0.25 | 2.90E-03 | 0.77 | 0.26 | 3.70E-02 |
| 395 | E2 | GSNTA**D**Q | 0.118 | 0.044 | 3.00E-03 | -1.09 | 0.37 | 3.80E-02 |
| 232 | E1 | D**E**NV | 0.176 | 0.084 | 3.60E-03 | -0.84 | 0.29 | 4.30E-02 |
| 580 | E2 | F**L**YI | 0.101 | 0.034 | 3.70E-03 | -1.18 | 0.41 | 4.40E-02 |
| 1429 | NS3 | **T**AS | 0.394 | 0.492 | 4.20E-03 | 0.64 | 0.22 | 4.80E-02 |
| 2470 | NS5B | **K**R | 0.878 | 0.953 | 4.50E-03 | 1.03 | 0.36 | 5.00E-02 |
| 2794 | NS5B | R**L**QH | 0.25 | 0.368 | 4.50E-03 | 0.62 | 0.22 | 5.00E-02 |
| 1635 | NS3 | IT**V** | 0.037 | 0.118 | 4.70E-03 | 1.21 | 0.43 | 5.10E-02 |
| 869 | NS2 | **S**G | 0.532 | 0.613 | 4.90E-03 | 0.59 | 0.21 | 5.20E-02 |
| 475 | E2 | **A** | 0.915 | 0.973 | 5.30E-03 | 1.25 | 0.45 | 5.40E-02 |
| 576 | E2 | NDS**G**K | 0.088 | 0.031 | 5.40E-03 | -1.21 | 0.43 | 5.40E-02 |
| 662 | E2 | **Q**L | 0.803 | 0.895 | 5.80E-03 | 0.75 | 0.27 | 5.60E-02 |
| 375 | E1 | **M**VIL | 0.691 | 0.811 | 6.10E-03 | 0.6 | 0.22 | 5.80E-02 |
| 500 | E2 | **S**LA | 0.383 | 0.502 | 6.10E-03 | 0.53 | 0.2 | 5.80E-02 |
| 438 | E2 | I**L** | 0.102 | 0.196 | 6.40E-03 | 0.78 | 0.29 | 6.00E-02 |
| 2799 | NS5B | K**R** | 0.197 | 0.105 | 6.70E-03 | -1.14 | 0.42 | 6.10E-02 |
| 577 | E2 | **S**T | 0.798 | 0.882 | 7.50E-03 | 0.69 | 0.26 | 6.40E-02 |
| 1764 | NS4B | **H**Y | 0.979 | 0.926 | 8.10E-03 | -1.5 | 0.57 | 6.90E-02 |
| 843 | NS2 | **M**I | 0.899 | 0.838 | 8.50E-03 | -0.9 | 0.34 | 7.00E-02 |
| 777 | P7 | **Y**H | 0.492 | 0.618 | 8.60E-03 | 0.61 | 0.23 | 7.00E-02 |
| 1498 | NS3 | **T** | 0.995 | 0.933 | 8.70E-03 | -2.74 | 1.05 | 7.00E-02 |
| 1807 | NS4B | **M**VLI | 0.83 | 0.73 | 9.10E-03 | -0.62 | 0.24 | 7.20E-02 |
| 781 | P7 | **K**R | 0.893 | 0.815 | 9.50E-03 | -0.75 | 0.29 | 7.40E-02 |
| 394 | E2 | **R**HQYSF | 0.382 | 0.509 | 9.50E-03 | 0.5 | 0.19 | 7.40E-02 |
| 442 | E2 | **F**IL | 0.77 | 0.861 | 9.70E-03 | 0.64 | 0.25 | 7.40E-02 |
| 881 | NS2 | **Y**H | 0.979 | 0.926 | 1.00E-02 | -1.44 | 0.56 | 7.60E-02 |
| 407 | E2 | A**P**S | 0.425 | 0.299 | 1.00E-02 | -0.51 | 0.2 | 7.60E-02 |
| 2361 | NS5A | **D** | 0.925 | 0.976 | 1.20E-02 | 1.2 | 0.48 | 8.40E-02 |
| 1196 | NS3 | **I**V | 0.904 | 0.963 | 1.20E-02 | 1.01 | 0.4 | 8.40E-02 |
| 2036 | NS5A | TA**S** | 0.186 | 0.131 | 1.20E-02 | -0.7 | 0.28 | 8.40E-02 |
| 388 | E2 | T**S**IV | 0.194 | 0.108 | 1.20E-02 | -0.67 | 0.27 | 8.60E-02 |
| 397 | E2 | SRY**Q**FHL | 0.027 | 0.092 | 1.30E-02 | 1.26 | 0.51 | 9.10E-02 |
| 387 | E2 | TV**I** | 0.254 | 0.156 | 1.30E-02 | -0.59 | 0.24 | 9.10E-02 |
| 299 | E1 | **Q** | 0.878 | 0.946 | 1.40E-02 | 0.84 | 0.35 | 9.50E-02 |
| 1979 | NS5A | **T**DV | 0.601 | 0.716 | 1.50E-02 | 0.52 | 0.22 | 9.50E-02 |
| 151 | C | **L**F | 0.877 | 0.946 | 1.50E-02 | 0.84 | 0.35 | 9.50E-02 |
| 873 | NS2 | V**A**I | 0.128 | 0.239 | 1.50E-02 | 0.65 | 0.27 | 9.50E-02 |
| 2489 | NS5B | **K**Q | 0.813 | 0.793 | 1.60E-02 | 1.94 | 0.8 | 9.50E-02 |
| 546 | E2 | S**G**NR | 0.08 | 0.155 | 1.60E-02 | 0.77 | 0.32 | 9.50E-02 |
| 558 | E2 | T**S** | 0.08 | 0.027 | 1.60E-02 | -1.09 | 0.45 | 9.70E-02 |
| 1993 | NS5A | T**A** | 0.085 | 0.03 | 1.70E-02 | -1.04 | 0.43 | 9.90E-02 |
