# Supplementary material for: Interferon lambda 4 impacts the genetic diversity of hepatitis C virus
Source: eLife. 2019 Sep 3;8:e42463. doi: 10.7554/eLife.42463 (PMC6721795; doi:10.7554/eLife.42463)
Supplement: Supplementary file 3. [file elife-42463-supp3.docx]

**Supplementary File 3**: *P*-value of Fisher’s exact test for enrichment or depletion of the association signals in HCV proteins and HLA restricted epitopes.

| Protein | *P*-value |
| --- | --- |
| C | 0.50 |
| E1 | 0.52 |
| E2 | 0.12 |
| P7 | 1 |
| NS2 | 1 |
| NS3 | 0.56 |
| NS4A | 1 |
| NS4B | 0.79 |
| NS5A | 0.53 |
| NS5B | 1 |
| HLA restricted epitope regions | 0.88 |
