# Supplementary material for: Interferon lambda 4 impacts the genetic diversity of hepatitis C virus
Source: eLife. 2019 Sep 3;8:e42463. doi: 10.7554/eLife.42463 (PMC6721795; doi:10.7554/eLife.42463)
Supplement: Supplementary file 4. — Note that the FDR was calculated independently for each viral GWAS against each host SNP. All the significant associations for each viral GWAS (against each of the 500 frequency matched host SNP) are shown in this table. [file elife-42463-supp4.docx]

**Supplementary File 4**: Associations between HCV amino acids and the 500 frequency-matched host SNPs at 5% FDR. Note that the FDR was calculated independently for each viral GWAS against each host SNP. All the significant associations for each viral GWAS (one against each of the 500 frequency matched host SNP) are shown in this table.

| SNP | Chromo-some | Position on Chromo-some | HCV amino acid position | Associated amino acid | *P* | q value |
| --- | --- | --- | --- | --- | --- | --- |
| rs3112346 | chr7 | 135702747 | 337 | I | 4.79E-05 | 4.67E-02 |
| rs3818048 | chr1 | 17071812 | 392 | V | 3.01E-05 | 2.94E-02 |
| rs910232 | chr1 | 17072019 | 392 | V | 3.01E-05 | 2.94E-02 |
| rs7792330 | chr7 | 138632991 | 399 | L | 1.57E-05 | 1.53E-02 |
| rs836116 | chr11 | 34547051 | 471 | P | 5.08E-05 | 4.77E-02 |
| rs6803628 | chr3 | 157407795 | 561 | V | 2.07E-05 | 1.79E-02 |
| rs2503712 | chr1 | 2205929 | 1267 | F | 4.26E-05 | 4.01E-02 |
| rs12152089 | chr21 | 21147102 | 1503 | A | 4.89E-05 | 4.71E-02 |
| rs2826674 | chr21 | 21133424 | 1503 | A | 4.33E-05 | 3.66E-02 |
