# Supplementary material for: Interferon lambda 4 impacts the genetic diversity of hepatitis C virus
Source: eLife. 2019 Sep 3;8:e42463. doi: 10.7554/eLife.42463 (PMC6721795; doi:10.7554/eLife.42463)
Supplement: Supplementary file 5. — We used logistic regression to test for association between host IFNL4 SNP (CC vs. non-CC) and codon changes. We included the first two viral and the first three host PCs as covariates. Only codons at which there were at least 20 synonymous and 20 non-synonymous codons for the most common codon at the site (348 codon sites across the HCV coding sequence) were included in the analysis. [file elife-42463-supp5.docx]

**Supplementary File 5**: Host *IFNL4* SNP rs12979860 association with changes from the most common codon to non-synonymous codons in HCV, at 10% FDR. We used logistic regression to test for association between host *IFNL4* SNP (CC vs. non-CC) and codon changes. We used the first two viral and three host PCs as covariate. Only codons at which there were at least 20 synonymous and 20 non-synonymous codons (348 codons across the HCV coding sequence).

| HCV amino acid position | HCV gene | Most common codon | Most common codon translation | *P* | q value |
| --- | --- | --- | --- | --- | --- |
| 2414 | NS5A | AGC | S | 2.82E-07 | 7.63E-05 |
| 2570 | NS5B | GTT | V | 3.79E-06 | 5.13E-04 |
| 60 | C | GGA | G | 2.42E-05 | 2.18E-03 |
| 940 | NS2 | AGC | S | 8.78E-05 | 5.94E-03 |
| 109 | C | CCA | P | 1.44E-04 | 7.82E-03 |
| 372 | E1 | GCT | A | 2.24E-04 | 1.01E-02 |
| 521 | E2 | GCT | A | 4.74E-04 | 1.83E-02 |
| 1740 | NS4B | ACC | T | 5.89E-04 | 1.99E-02 |
| 457 | E2 | AGC | S | 8.78E-04 | 2.64E-02 |
| 2567 | NS5B | GTG | V | 1.41E-03 | 3.55E-02 |
| 1975 | NS5A | GAT | D | 1.44E-03 | 3.55E-02 |
| 349 | E1 | GCC | A | 2.03E-03 | 4.45E-02 |
| 1158 | NS3 | CTC | L | 2.15E-03 | 4.45E-02 |
| 1429 | NS3 | ACA | T | 2.30E-03 | 4.45E-02 |
| 402 | E2 | CTT | L | 2.57E-03 | 4.64E-02 |
| 500 | E2 | TCA | S | 2.79E-03 | 4.73E-02 |
| 1024 | NS2 | CGT | R | 3.28E-03 | 5.17E-02 |
| 232 | E1 | GAC | D | 3.44E-03 | 5.17E-02 |
| 475 | E2 | GCT | A | 3.94E-03 | 5.61E-02 |
| 777 | P7 | TAC | Y | 4.35E-03 | 5.89E-02 |
| 479 | E2 | GGT | G | 5.33E-03 | 6.61E-02 |
| 2955 | NS5B | AAT | N | 5.44E-03 | 6.61E-02 |
| 577 | E2 | TCA | S | 5.61E-03 | 6.61E-02 |
| 524 | E2 | GCC | A | 6.37E-03 | 7.19E-02 |
| 580 | E2 | TTC | F | 6.86E-03 | 7.23E-02 |
| 394 | E2 | CGT | R | 6.95E-03 | 7.23E-02 |
| 442 | E2 | TTT | F | 7.38E-03 | 7.40E-02 |
| 399 | E2 | TTT | F | 8.37E-03 | 7.79E-02 |
| 1764 | NS4B | CAT | H | 8.43E-03 | 7.79E-02 |
| 781 | P7 | AAA | K | 8.63E-03 | 7.79E-02 |
| 1498 | NS3 | ACG | T | 9.21E-03 | 7.89E-02 |
| 2361 | NS5A | GAC | D | 9.32E-03 | 7.89E-02 |
| 1416 | NS3 | GCG | A | 1.05E-02 | 8.61E-02 |
| 2320 | NS5A | GGC | G | 1.09E-02 | 8.68E-02 |
| 1962 | NS4B | CAG | Q | 1.15E-02 | 8.92E-02 |
