# Supplementary material for: Interferon lambda 4 impacts the genetic diversity of hepatitis C virus
Source: eLife. 2019 Sep 3;8:e42463. doi: 10.7554/eLife.42463 (PMC6721795; doi:10.7554/eLife.42463)
Supplement: Supplementary file 6. — We used logistic regression to test for association between host IFNL4 SNP (CC vs. non-CC) and codon changes. We included the first two viral and the first three host PCs as covariate. Only codons at which there were at least 20 synonymous and 20 non-synonymous codons for the most common codon at the site (348 codon sites across the HCV coding sequence) were included in the analysis. [file elife-42463-supp6.docx]

**Supplementary File 6**: Host *IFNL4* SNP rs12979860 association with changes from the most common codon to synonymous codons in HCV, at 10% FDR. We used logistic regression to test for association between host *IFNL4* SNP (CC vs. non-CC) and codon changes. We used the first two viral and three host PCs as covariate. Only codons at which there were at least 20 synonymous and 20 non-synonymous codons (348 codons across the HCV coding sequence).

| HCV amino acid position | HCV gene | Most common codon | Most common codon translation | *P* | q value |
| --- | --- | --- | --- | --- | --- |
| 1416 | NS3 | GCG | A | 2.37E-05 | 7.95E-03 |
| 2333 | NS5A | ATT | I | 7.09E-05 | 1.19E-02 |
| 1024 | NS2 | CGT | R | 7.85E-04 | 7.73E-02 |
| 624 | E2 | TTT | F | 9.20E-04 | 7.73E-02 |
