# Supplementary material for: Interferon lambda 4 impacts the genetic diversity of hepatitis C virus
Source: eLife. 2019 Sep 3;8:e42463. doi: 10.7554/eLife.42463 (PMC6721795; doi:10.7554/eLife.42463)
Supplement: Supplementary file 7. [file elife-42463-supp7.docx]

**Supplementary File 7**: *IFNL4* haplotype combination and predicted protein for host SNPs rs117648444 and rs368234815 in the EAP (N=74) and BOSON (N=411) cohorts and in the combined cohort (N=485).

| BOSON |  |  | | | | |
| --- | --- | --- | --- | --- | --- | --- |
| Protein | IFN-λ4-Null | IFN-λ4-P70 | | | IFN-λ4-S70 | |
| Haplotypes | TT/G  TT/G | TT/G  ΔG/G | ΔG/G  ΔG/G | ΔG/G  ΔG/A | TT/G  ΔG/A | ΔG/A  ΔG/A |
| Counts | 145 | 167 | 30 | 21 | 42 | 6 |
| Proportion | 0.35 | 0.41 | 0.07 | 0.05 | 0.10 | 0.01 |

| EAP |  |  | | | | |
| --- | --- | --- | --- | --- | --- | --- |
| Protein | IFN-λ4-Null | IFN-λ4-P70 | | | IFN-λ4-S70 | |
| Haplotypes | TT/G  TT/G | TT/G  ΔG/G | ΔG/G  ΔG/G | ΔG/G  ΔG/A | TT/G  ΔG/A | ΔG/A  ΔG/A |
| Counts | 41 | 19 | 4 | 3 | 7 | 0 |
| Proportion | 0.55 | 0.26 | 0.05 | 0.04 | 0.09 | 0 |

| BOSON +EAP |  |  | | | | |
| --- | --- | --- | --- | --- | --- | --- |
| Protein | IFN-λ4-Null | IFN-λ4-P70 | | | IFN-λ4-S70 | |
| Haplotypes | TT/G  TT/G | TT/G  ΔG/G | ΔG/G  ΔG/G | ΔG/G  ΔG/A | TT/G  ΔG/A | ΔG/A  ΔG/A |
| Counts | 186 | 186 | 34 | 24 | 49 | 6 |
| Proportion | 0.384 | 0.384 | 0.07 | 0.05 | 0.10 | 0.012 |
